# Multi Omics Applications in Biological Systems

**DOI:** 10.3390/cimb46060345

**Published:** 2024-06-11

**Authors:** Cristian D. Gutierrez Reyes, Gerardo Alejo-Jacuinde, Benjamin Perez Sanchez, Jesus Chavez Reyes, Sherifdeen Onigbinde, Damir Mogut, Irma Hernández-Jasso, Denisse Calderón-Vallejo, J. Luis Quintanar, Yehia Mechref

**Affiliations:** 1 Department of Chemistry and Biochemistry, Texas Tech University, Lubbock, TX 79409, USA; sonigbin@ttu.edu; 2 Department of Plant and Soil Science, Institute of Genomics for Crop Abiotic Stress Tolerance (IGCAST), Texas Tech University, Lubbock, TX 79409, USA; galejoja@ttu.edu (G.A.-J.); bperezsa@ttu.edu (B.P.S.); 3 Center of Basic Sciences, Department of Physiology and Pharmacology, Autonomous University of Aguascalientes, Aguascalientes 20392, Mexico; jesus.chavezr@edu.uaa.mx (J.C.R.); irma.hernandez@edu.uaa.mx (I.H.-J.); denisse.calderon@edu.uaa.mx (D.C.-V.); jlquinta@correo.uaa.mx (J.L.Q.); 4 Department of Food Biochemistry, Faculty of Food Science, University of Warmia and Mazury in Olsztyn, 10-719 Olsztyn, Poland; damir.mogut@uwm.edu.pl

**Keywords:** systems biology, omics, genomics, transcriptomics, proteomics, glycoproteomics, glycomics, metabolomics, lipidomics, foodomics

## Abstract

Traditional methodologies often fall short in addressing the complexity of biological systems. In this regard, system biology omics have brought invaluable tools for conducting comprehensive analysis. Current sequencing capabilities have revolutionized genetics and genomics studies, as well as the characterization of transcriptional profiling and dynamics of several species and sample types. Biological systems experience complex biochemical processes involving thousands of molecules. These processes occur at different levels that can be studied using mass spectrometry-based (MS-based) analysis, enabling high-throughput proteomics, glycoproteomics, glycomics, metabolomics, and lipidomics analysis. Here, we present the most up-to-date techniques utilized in the completion of omics analysis. Additionally, we include some interesting examples of the applicability of multi omics to a variety of biological systems.

## 1. Introduction

Omics is an interdisciplinary research field focused on understanding the interactions within biological systems from a holistic perspective, considering the entire system rather than individual parts [1,2]. This field emphasizes the use of high-throughput technologies and large-scale data generated at the molecular level. Computational tools are then employed to integrate and analyze the data, enabling an understanding of biological processes. The “omics” concept encloses the subfields of genomics [3], transcriptomics [4], proteomics [5], glycoproteomics [6], glycomics [7], metabolomics [8], lipidomics [9], and several others, providing a comprehensive analysis of the corresponding biomolecules.

**Figure 1:**
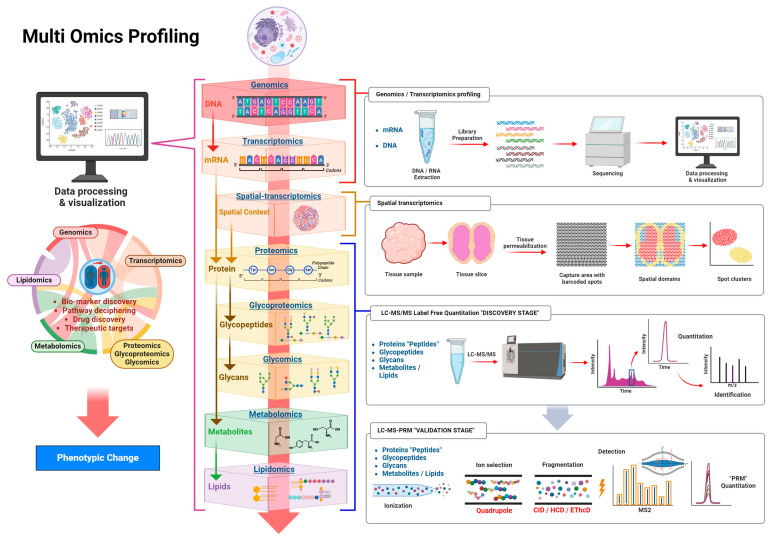
Multi omics. Inside the eukaryotic nucleus, spatial conformations of genomic material and interactions participate in regulating gene expression. Different RNAs are accumulated, and some splice variants are produced in response to internal and external stimuli. Transcripts can be rapidly degraded or preserved and can sustain high or low translation rates. Post translational modifications provide different protein functions that can modify ligand affinity, protein interactions, catalytic activity, etc. The cellular regulatory processes favor synthesis, degradation, modification, and interaction of a multitude of molecules. The right section of the figure shows high-throughput sequencing methods and technologies to measure gene expression in situ, and the common LC-MS/MS label-free quantitation “discovery stage” followed by the targeted “validation stage”.

In recent years, omics science has emerged as a pivotal area of research in understanding cellular and molecular systems, significantly enhancing our comprehension of human health, development, diagnosis, prognosis, and aging [10]. Omics technologies offer detailed insights by cataloging variations linked with different stages of diseases or a variety of biological processes, thereby serving as a critical tool for monitoring disease progression and developing biomarkers. Stand-alone omics approaches offer a restricted point of view of the studied system, offering insights into specific molecules and their potential significance in metabolic pathways. Additionally, these approaches lack the necessary information for a comprehensive understanding of dynamic biological processes. In this regard, multi omics plays a central role in unraveling the relationships between different biomolecules and their interactions by simultaneously examining different single omics approaches (Figure 1), to provide a holistic view of the biological system [11]. This allows for the validation of individual findings and reduces the risk of false positives. As a fundamental component of system biology, multi omics allows researchers to explore biological systems as interconnected networks. However, the integration of omics data is challenging due to factors such as large data sets, data heterogeneity, sample sizes, and the need for advanced statistical methods [1]. Multi omics approaches could shed light on the fundamental causes of diseases, their functional repercussions, and pertinent interactions [12,13]. Together, omics technologies offer a comprehensive view of the biological status and changes occurring within an organism, especially in the context of disease. The integration of data from these diverse sources allows researchers to construct a more comprehensive model of disease mechanisms. This integrated approach helps identify potential diagnostic markers and therapeutic targets and enhances our understanding of the complex network of biological pathways involved in disease etiology and progression. By enabling a systems-level analysis, omics sciences facilitate the identification of key regulatory nodes and pathways that could be targeted for intervention, paving the way for personalized medicine and improved healthcare outcomes [14,15,16].

This review provides a comprehensive and up-to-date description of the most widely used omics techniques and describes their applicability for understanding biological systems. Additionally, our review focuses on holistic approaches that have demonstrated the benefits of multi omics applications.

## 2. Genomics and Transcriptomics

A few decades ago, scientists studied single genes at a time. Current technological innovations allow fast and cost-effective sequencing of entire genomes [17]. This tremendous change in sequencing capability is reflected in the first human genome, which was produced by an international consortium that cost billions of dollars; today, the sequencing of individual genomes can be completed for a reasonable price in a relatively short time [18]. Such technological progress encourages research efforts focused on genomic studies rather than single genes. Genomics encompasses a wide range of approaches to study and understand organisms’ DNA. This includes the analysis of genome structure and composition (genes as well as non-coding regions), their functions and interaction with each other, variation, evolution, and more. The significant improvement in the throughput and accuracy of sequencing capabilities has had an important impact on the identification of novel coding and regulatory regions [19], as well as on our understanding of the spatial organization of the DNA within a cell nucleus [20]. Genomics has also revolutionized the pharmaceutical industry by allowing the identification of protein targets and accelerating drug discovery [21,22,23]. The field has seen rapid expansion, with models and methods developed for analyzing genome rearrangements [24], gene duplication, phylogenetic networks [25], and gene clustering [26].

While genomics provides an overview of all the genetic information of organisms, transcriptomics refers to the study of gene expression patterns of the entire set of RNA molecules in an individual [27]. High-throughput sequencing technologies (e.g., Illumina) allow quantifying the number of times a given sequence is present in a sample. In global transcriptomics analyses, RNA is isolated from tissue samples and further prepared to enrich messenger RNAs (mRNAs), small RNAs (sRNAs), long non-coding RNAs (lncRNA), etc. The resulting data represent a snapshot of the enriched RNA abundance from the bulk of cells within the sample. These analyses provide an average representation of gene expression across a population of cells [28]. Although transcriptomics offers an important source of information, it does not tell the whole story about transcripts. It does not consider the rate at which transcripts are produced or degraded, nor how much of the transcripts are translated into proteins. Additional techniques, such as polysome profiling, allow researchers to estimate the translation rate of a transcript by measuring the number of ribosomes attached to individual mRNAs [29,30], and ribosome profiling can tell the ribosome footprint by isolating the section of the transcript that is bound to the ribosome [31,32]. More sophisticated library preparations involving reversible inhibition of transcription elongation combined with tagging nascent RNA can also provide information about the specific transcription rates of mRNAs and their degradation, as well as providing clues about the splicing process [33,34,35]. More recently, long-read sequencing techniques, such as PacBio Iso-Seq or Oxford Nanopore direct RNA-seq, allow the sequencing of complete transcripts to detect multiple isoforms from the same gene and their dynamics between distinct treatments [36].

Multicellular organisms show a great diversity in cellular composition. For instance, even though a human being originates from a single diploid cell as tissue develops, different cells fall into a heterogeneity of cellular fates influenced by internal and external factors [37,38]. Moreover, once fully developed, cells from the same tissues undergo distinct tasks. Currently, platforms such as Fluidigm C1, Clontech iCell8, BD Rhapsody, and 10× Genomics Chromium allow the isolation of individual cells or nuclei to prepare single-cell RNA-seq (scRNA-seq) libraries [39]. Every platform has its strengths and limitations. A recent study compared the droplet-based 10× Chromium and the microwell-based BD Rhapsody [40]. The results of this study indicate that the microwell-based platform exhibits better mRNA capture efficiency, but it can be under-represented or even lose larger cell types compared to the droplet-based system. The scRNA-seq technology has been applied to understand the dynamic activities of transcription regulation over time and the trajectories of cell states at the individual cell level, for instance, in response to neuronal activation [41]. Moreover, generating multiple single-cell data has allowed scientists to produce reference single-cell atlases to compare cellular conditions across various research investigations [42]. Although scRNA-seq has significantly increased our understanding of the complex cellular heterogeneity of tissues and organs, it has some disadvantages, such as the effect of cell dissociation and the loss of positional information [43]. The spatial context of the cells has a significant role in developmental biology, cellular communication, and disease study, among others. Emerging technologies to determine cell gene expression preserving in situ spatial locations within a tissue are known as spatial transcriptomics (ST). These technologies can be broadly divided into imaging-based and sequencing-based methods [44]. Imaging-based technologies rely on fluorescence in situ hybridization of probes that target specific genes. Conversely, sequencing-based technologies depend on spatial barcodes on an array to capture transcripts unbiasedly. Recent reviews have discussed currently available ST technologies’ characteristics, applications, advantages, and limitations [45,46]. Commercially available ST technologies for whole transcriptome profiling include the 10× Visium, which resolves several to dozens of cells [47]. New methods such as Stereo-seq can provide single-cell resolution, and it has been successfully used to study the spatiotemporal transcriptomic dynamics during organogenesis [48].

## 3. Proteomics, Glycoproteomics, and Glycomics

Proteomics and its associated glycoproteomics and glycomics techniques are dynamic research areas working to elucidate the associations between protein structure and function [49,50,51]. Protein abundance and post-translational modifications are the fundamental parameters utilized to evaluate the dynamic behavior of proteins. The exponential growth of these omics was possible due to their integration with mass spectrometry-based (MS-based) analysis, which enormously contributed to the creation of large and reliable data sets from a variety of sample types such as plants [52], animal models, human bodily fluids, tissues, etc. [53,54,55,56].

In MS-based proteomics, the proteins are identified using two common approaches: bottom up and top down. In the bottom-up approach, the intact proteins are digested into peptides prior to introduction to the mass spectrometer, where they are detected and fragmented to facilitate identification [57]. In top-down proteomics, the proteins are ionized and fragmented in the intact form in the mass spectrometer [58]. This approach allows an important characterization of protein isoforms and provides a general picture of the protein’s post-translational modifications, such as glycosylation, acetylation, and phosphorylation [59,60]. The protein purification stages are crucial for the obtention of sensitive and reproducible results. In the past decade, proteomics analysis was commonly performed on bodily fluids such as serum, plasma, and cerebrospinal fluid (CSF) and different types of tissues [61,62,63]. These investigations mainly searched for abundance changes in the proteome of disease samples compared to healthy controls. At present, proteomics analysis has extended to more complicated samples such as extra vesicular exosomes (EVs) [64], nucleosomes [65], or single-cell approaches [66]. For instance, Van den Ackerveken et al. [65] developed a fast and robust enrichment method able to isolate nucleosomes from plasma. Further top-down proteomics analysis of colorectal cancer (CRC) samples showed alterations in the histone PTMs after alterations of epigenetic modification [65,67]. The approach was able to identify and quantify 13 histone PTMs with significant changes in abundance between the CRC and the control samples. Recent advancements in MS instruments, such as the development of the Orbitrap Astral (Thermo Fisher Sci.), allowed researchers in the field to address single-cell proteomics using label-free quantitative (LFQ) analysis. Ye et al. [68] developed a lossless LFQ single-cell proteomics (SCP) method used for the identification of over 40,000 peptides and 5000 proteins in Hela cells. The approach integrated sample preparation utilizing the cellenONE, the proteoCHIP EVO96, and the direct transference of the sample to Evotip disposal trap columns. The samples were subsequently analyzed using the Evosep One LC, with whisper flow gradients linked to a narrow-window data independent acquisition (DIA) system.

Glycoproteomics and glycomics MS-based analysis is dedicated to the comprehensive study of carbohydrate moieties present in cells and organisms. These branches of omics science delve into understanding the structure, function, and dynamics of glycans attached to proteins and lipids across different biological systems and their role in health and disease. By analyzing these structures, glycomics and glycoproteomics analysis provides insights into cellular communication [69,70], immune response [71], pathogen interaction [72,73], and disease progression [74], making it a crucial field in biomedical research and therapeutic development. Advanced technologies such as high-performance liquid chromatography–mass spectrometry (LC-MS) are employed to decipher the complex and diverse structures of glycans and the protein *O*- and *N*-site heterogeneity. Derivatization techniques are essential due to the low ionization efficiency and poor stability of native glycan and glycopeptide structures that hinder their accurate quantitation and detection by MS systems [75,76,77]. Through derivatization, researchers can achieve more accurate glycan and glycopeptide profiling, facilitating a deeper understanding of glycan functions in biological processes and disease mechanisms. Common glycan derivatization techniques include hydrazide chemistry [78], carbamate derivatization [79], chemical labeling by reductive amination [80], and permethylation [81]. In the case of glycopeptides, the glycan moiety can be derivatized through selective reductive amination reactions directed to the sialic acid [76,77]. The glycopeptides can also be derivatized, targeting the peptide *N*-terminus using tandem mass tags (TMTs) [82]. Alterations in glycan expression are linked to various diseases, including cancer [83,84,85], neurodegenerative diseases [86,87,88], autoimmune disorders [89,90], and infectious diseases [73,91,92]. These changes can impact cell signaling [93], immune recognition [94], and molecular stability [95], leading to altered cellular behaviors and disease progression. For example, in cancer, altered glycosylation can promote tumor growth, facilitate metastasis, and enable evasion of immune surveillance [96]. In autoimmune diseases, aberrant glycosylation can lead to improper immune responses against self-antigens [90]. Understanding these changes is crucial for developing diagnostic markers and therapeutic targets. Integrating glycomics and proteomics analyses enhances our understanding of disease mechanisms by providing a comprehensive view of the glycome and its interaction with proteins [97,98,99]. Mechref and co-workers [53] have shown that integrating proteomics and glycomics analysis can shed light on the glycoprotein source of glycans and provide mechanistic insight into the clinicopathological changes observed in different disease conditions [53]. This strategy was applied to samples derived from healthy and mild cognitive impairment (MCI) patients. The results pointed to the isomeric *N*-glycans GlcNAc_5_,Hex_6_,Neu5Ac_3_ and GlcNAc_4_,Hex_5_,Fuc,Neu5Ac as biomarker candidates to differentiate healthy and MCI patients. Moreover, the proteomics analysis showed a correlation of the identified glycoproteins with neuroinflammation, an important process in the progression of brain-related diseases. The same research group also utilized proteomics-glycomics approaches to describe the protein glycosylation differences in 11 SARS-CoV2 variants. A significantly large abundance of sialofucosylated *N*-glycans was observed in the variants of concern when compared to the variants of interest. The proteomics analyses were used for the validation of variant amino acid sequences [92].

## 4. Metabolomics and Lipidomics

The metabolome includes all the small-molecule metabolite parts of a biological sample, including structural functions, stimulatory and inhibitory effects, fuel storage molecules, and others [100]. Lipids are structurally diverse molecules that perform multiple important functions within the cell. They are characterized by their hydrophobic nature and fundamental components of the cellular membranes [101]. Either the metabolome or the lipidome can be studied at the single-cell level or in a more complex matrix such as a bodily fluid or an organ tissue [100,102,103]. The analyses of these biological systems using high-throughput targeted or untargeted metabolomics or lipidomics MS-based analysis provide an invaluable opportunity to study and quantify differential abundance [104].

Recently, a few techniques based on single-cell metabolomics (SCM) have been developed, including Patch clamp-based nano ESI-MS, CE-MS-based SCMS, MALDI-MSI-based SCMS, LALDI-MSI-based SCMS, SIMS-MSI-based SCMS, and mass cytometry-based SCMS [105]. Metabolites are crucial components of biochemical pathways and cellular functions; therefore, quantitative metabolomics has become a powerful tool in many research fields, including those investigating cancer and neurodegenerative disorders. In this sense, there is a challenge to explore and identify metabolic hallmarks that can track disease development and progression [106]. Cancer cells can reprogram their metabolic pathways to adapt tumor microenvironments and survive pharmacological treatments [107]. This phenomenon, known as metabolic plasticity, results in a heterogenous tumor with different metabolic phenotypes that are hard to characterize and consequently difficult to treat [108]. In this regard, SCM has been applied in the identification of sub-types of circulating tumor cells with distinct metastatic potential [109], and provided useful information for drug therapy [110,111]. In addition, SCM has been used to study the dynamic cancer cell–cell interaction during the drug resistance process [112] and the potential role of microbiota in the development and progression of different cancer types [113]. As metabolites directly reflect the cell behavior, the integration of SCM with other single-cell omics could suggest mechanisms underlying tumor development and drug resistance. Metabolomics have also been used to investigate neurodegenerative disorders [114]. Despite the expanding prevalence of SCM, scarce evidence of studying neurodegenerative disorders at the single-cell level has been reported. Metabolomics was applied to a preclinical model of Alzheimer’s disease (AD); seven AD-related neurotransmitters and sixteen biomarkers were identified by SCM in PC12 cells [115]. It is only a matter of time before SCM will be employed on neurodegenerative disorders to explore the individual role of each neuron and glia cell in the progress of neurodegeneration.

Other most affordable LC–MS/MS strategies employed for metabolomics analysis of biological samples have served to identify potential biomarkers in human serum from patients with diabetes [116], schizophrenia [117], ischemic stroke [118], and other health conditions [119,120,121]. Similar technique also have been used to assess food quality [122,123,124,125,126] and the toxic effects of xenobiotics [127,128]. In order to enhance accuracy, precision, and throughput, common LC-MS techniques have employed ^13^C or ^18^O isotopic approaches [129,130,131,132]. MALDI imagining is an MS-based technique widely used for the characterization of tissues derived from several health conditions facilitating their diagnosis and therapy [133,134,135,136,137].

Lipids possess significant nutritional value due to their high caloric density and their capacity to transport essential substances that the human body cannot produce independently. Lipids are also involved in intra- and extracellular signaling processes [138]. Lipidomics focuses on the study and characterization of cellular and bodily fluid lipids, along with their interactions and functions within the body. This field is a crucial tool in healthcare for identifying biomarkers useful in diagnosing and developing therapies for various diseases [139,140]. For example, the nervous system contains a large amount of lipids. It has been suggested that neurological disorders may be associated with lipid homeostasis, among other factors [103,141]. It has also been described that there are changes in the lipidomic profiles of bioactive lipids in plasma and CSF, as well as in specific anatomical regions of the brain associated with the pathological characteristics of AD and Parkinson’s disease [142]. Significant alterations in plasma lipids have also been described in rats and mice with spinal cord and sciatic nerve injury, which indicates that lipid metabolism could be related to the recovery and/or damage processes following nerve injury [143,144]. The application of lipidomics to disorders associated with metabolic syndrome has been widely described. Lipidomics plays a key role in risk prediction studies and therapeutic monitoring of diseases related to metabolic syndrome, given the close association of lipids with these diseases [145]. Furthermore, this discipline has been valuable for determining population profiles, conducting research on pathogenesis, and for identifying biomarkers and monitoring therapeutic responses [146]. It has been described that the lipidome is altered in many neoplastic diseases. The main applications of lipidomics in these types of disorders have focused on the detection and classification of neoplastic cells or tissues, on differentiation between neoplastic and normal environments, in the evaluation of cancer treatments, and for the discovery of new tumor biomarkers [147].

## 5. Multi Omics Integration

The study of organisms as complex systems requires the integration of distinct approaches (Figure 1). For instance, although genomic and transcriptomic analyses offer powerful tools in our understanding of molecular processes, downstream gaps cannot be filled without integrating other approaches. None of the above methods provide information about what happens to a gene’s translated product or the effect of metabolite accumulation. Consideration of these questions highlights the relevance of multi omics analysis, as they provide a more complete and holistic landscape of cell molecular interactions. Table 1 summarizes additional omics approaches, focusing on the multi-integration of molecular data sets.

Omics technologies are high-throughput molecular assays that produce large data sets of information. Public repositories facilitate the accessibility of this information to the scientific community. Some examples are the following: for high-throughput sequencing SRA [173]; for functional genomics and DNA sequence information NCBI GEO [174], GenBank [175]; for spatial transcriptomics CROST [176]; for proteomics and glycoproteomics PRIDE [177], Proteome Xchange [178]; for glycomics GlycoPOST [179]; for metabolomics and lipidomics MetaboLights [180], HMDB [181]; and many others for multi omics as listed in the references [182,183,184]. However, single omics relay in the application of common statistics mainly focusing on the differentiation of the studied groups, for example, principal component analysis (PCA) plots; heat maps; receiver operating characteristic (ROC) curves; dot plots; statistical tests such as Anova, t-test, Mann–Whitney U Test, and several others in accordance with the data size and distribution; and the appropriated false discovery rate (FDR) corrections such as Benjamini-Hochberg, Bonferroni, etc. [185,186,187]. Regarding the multi omics integration, the application of computational approaches is necessary due to the data complexity. Ingenuity pathway analysis (IPA) is an extensive database from QIAGEN Co. able to integrate transcriptomics, proteomics, and metabolomics data in diverse biological pathways [188]. A similar free alternative is iPathwayGuide developed from ADVAITA Co. [189]; many other software developments and its applications in diverse multi omics investigations are described in the following references: IOAT [190], multi omics of primary immunodeficiencies [191], Omics TIDE [192], IBAG [193], cancer omics [194], and TiMEG [195].

### Foodomics

Foodomics represents the integration of advanced omics technologies in food and nutrition studies, aimed at enhancing consumer welfare, health, and understanding [196]. This interdisciplinary field merges food chemistry, biological sciences, and data analysis techniques to comprehend food and its constituents at a molecular level, with the overarching goal of improving food quality, safety, and nutrition by exploring the intricate interactions between food components and the human body. In foodomics research, a primary focus lies in understanding the interplay between food constituents and biological systems, striving to identify biomarkers that objectively measure food intake and shed light on its physiological effects [197]. Foodomics methodologies find application in various studies aimed at linking dietary patterns with health outcomes. Research outcomes can utilize metabolomics techniques to identify specific compounds in diets and analyze their impact on health. For instance, ongoing studies apply metabolome analysis to unveil food-specific compounds (FSCs) that can bridge dietary patterns, such as the Mediterranean-style (MED) diet, with overall health outcomes. Drawing from a controlled feeding MED intervention, the study validates the identification of FSCs from eight distinct foods, detectable in biospecimens post-consumption [198]. Another example is the use of nuclear magnetic resonance (NMR) to reveal alterations of protein and carbohydrate metabolisms in different diets [199]. This study provides new insights into the effects of a healthy diet on glycemia, reduction of inflammation, and weight loss among obese individuals, as well as alteration of the gut microbiota metabolism.

Dilmore et al. [200] evidenced that the modified Mediterranean ketogenic diet (MMKD) could offer potential benefits in combating memory decline associated with AD. The study implemented MMKD dietary interventions for participants with mild cognitive impairment (MCI) to assess their cognitive function. The study utilized shotgun metagenomics to analyze the gut microbiota composition of participants, providing insights into how these dietary interventions influenced the microbiome. Additionally, untargeted metabolomics was employed to analyze metabolite profiles in samples collected at various intervals during the dietary interventions, to identify changes in metabolites related to cognitive function and gut health. However, the untargeted metabolomics platform has limitations in characterizing lipid metabolism and mitochondrial function compared to targeted platforms. Through metagenomic and metabolomic analyses, potential biomarkers associated with cognitive function and gut health were identified. For example, changes in the levels of GABA-producing microbes and GABA-regulating bacteria were observed in individuals with MCI following the MMKD, suggesting a link between gut microbiota composition and cognitive status. Overall, the study integrated a multi omics approach to investigate the relationship between dietary interventions, gut microbiota composition, metabolite profiles, and cognitive function in individuals at risk for AD.

Foodomics represents a dynamic and interdisciplinary field that holds promise for enhancing our understanding of food and its effects on health and well-being. This field has implications for food industry practices, public health policies, and personalized nutrition strategies, especially with the rise of alternative sources of nutrients.

## 6. Concluding Remarks

Omics sciences stand at the forefront of biomedical research, offering unprecedented insights into the molecular underpinnings of diseases. Their application in disease study promises to revolutionize the diagnosis, treatment, and prevention of human diseases, highlighting their indispensable role in advancing medical science and improving patient care. In this review, we have discussed the current technological innovations that allow fast and cost-effective sequencing of entire genomes. Additionally, we revised a set of novel and accurate transcriptomics techniques focused on showing how the transcript patterns are affected by the development or disease progression. The development of modern MS techniques has allowed the acquisition of high-throughput data sets in the proteomics, metabolomics, and lipidomics fields. Additionally, we described examples of recent studies using single cells or derived from diverse bodily fluids, as well as plant materials. This review offers an extensive and up-to-date overview of the most widely used omics techniques, detailing the relevance of the omics field in understanding different types of biological systems using a holistic view.

## Figures and Tables

**Table 1:** Complemental integrative omics applications.

Omics Spotlight	Omics Integration	Applications	References
Diagnosis and prognosis	G+T+P+M	Alzheimer’s disease diagnosis	[148]
	T+Me+scT	Evaluation of Covid-19 prognosis	[149]
	G+T+P+pP	Hepatocellular carcinoma classification	[150]
	G+T	Identification of prognostic biomarkers for gastric cancer	[151]
	P+M+L	Search for pathway alterations in Alzheimer’s disease	[152,153]
	G+T+M	Identification of metabolite biomarkers for predicting radiation resistance	[154]
	T+P+E	Epigenetic alterations associated with Alzheimer’s disease	[155]
	G+T+P	Gastrointestinal microbiome and diabetes mellitus (type 1)	[156]
	G+P+M	Liver disease pathogenesis	[157]
	T+P+M	Effect of heavy-metal exposure in neurodevelopment	[158]
	sT+sM	Spatial resolution approaches to study brain injuries	[159]
	sT+sM	Multimodal spatial approach used in Parkinson’s disease	[160]
	scT+E	Identification of genomic variants associated with eye diseases	[161]
Biomarker	T+M+L	Progression of lung fibrosis	[162]
	G+T+P	Metabolic mapping of Alzheimer’s disease	[163]
	G+T+M+Me	Identification of osteoporosis biomarkers	[164]
	M+L	Predictive biomarkers for COVID-19 severity	[165]
	G+T+P+pP	Exploration for Hepatocellular carcinoma biomarkers	[150]
	P+Gly	Identification of mild cognitive impairment biomarkers	[53]
	T+P	Temporal lobe epilepsy biomarkers	[166]
	T+P	Emphysema biomarkers	[167]
	T+P	Idiopathic pulmonary fibrosis	[168]
Drug targets and therapeutics	G+T+P	Alzheimer’s drug discovery	[169]
	G+T+P	Prostate cancer diagnosis and therapies	[170]
	P+Gly	Glycosylation profile of the S1 protein of eleven SARS-CoV-2 variants	[92]
	G+T	Drug response profiling of childhood acute lymphoblastic leukemia cell lines	[171]
	G+T	Prioritization of therapeutic targets for dyslipidemia	[172]

Abbreviations: G, genomics; T, transcriptomics; P, proteomics; M, metabolomics; L, lipidomics; Gly, glycomics; sc, single cell; p, phospho; Me, methylomics; E, epigenomics; and s, spatial.
